# S. Jasanoff (ed.): Reframing Rights: Bioconstitutionalism in the Genetic Age: Cambridge MA: MIT Press, 2011

**DOI:** 10.1186/2195-7819-9-6

**Published:** 2013-07-10

**Authors:** Atina Krajewska

**Affiliations:** Lecturer in Law, Cardiff Law School, Law Building Museum Avenue, Cardiff, CF10 3AX UK

**Keywords:** Genetics, Constitutionalism, Coproduction, Human rights, DNA, Embryo research

Shelia Jasanoff’s new edited volume on bioconstitutionalism provides an innovative and conceptually coherent analysis of the dynamic relationship between new technologies and constitutional frameworks. It is a most welcome introduction to a topic that requires extensive legal study and interdisciplinary enquiry. We learn from the *Acknowledgments* that the collection has been developed by academics affiliated with the Programme on Science, Technology, and Society (STS) at the J.F. Kennedy School of Government at the Harvard University. ^a^ It is indeed most telling that recent developments in life sciences and law have been defined in terms of constitutional theory by sociologists, anthropologists, bioethicists, and lawyers through the lenses of STS studies. ^b^ Despite an impressive body of legal writing analysing the effects of the biotechnological revolution on notions of legal personhood, subjectivity, human rights, and legitimacy, a comprehensive conceptualisation in constitutional theory is still lacking. This might stem from the fact that medical law, which deals with these issues more directly, has been traditionally associated with private rather than public law, and developed as a distinct category. Although exploring the reasons of such a development would be most interesting, it exceeds the scope of this book review. Although legal scholars – initially influenced by deterministic and essentialist views of genetics (Rothstein 2005, Rouvroy 2008, Huang 2000, Calvert 2008) – have been inventive in formulating new rights, including the right to genetic privacy (Gostin 1995, Dworkin 2000, Laurie 2002) reproductive autonomy, biological origins, or indeed ‘(gen-) informational self-determination’ (Enquete-Kommission des Deutschen Bundestag 2002; Stümper , Stümper Stümper 1996) traditionally the legislative or judicial acknowledgement of these rights at the constitutional level has been slow and scarce. Consequently, theorisations have been fragmented, usually focusing on particular issues, e.g. reproductive medicine, stem cell research, genetic testing, or biobanks. Jasanoff’s edited collection aims at addressing this gap. Therefore, it should interest a variety of academic audiences in the field of science and technology studies, bioethics, sociology, and most importantly law. Due to its comparative aspect, it could also be of interest to more inquisitive policy makers regulating life sciences.

The volume comprises twelve chapters which constitute case studies covering a variety of topics ranging from sterilisation, genetic testing, biobanks, stem cell and genomic research, to xenotransplantation and GMOs. They analyse different aspects of the constitutional design affected by the changes in life sciences, including the (re)construction of legal personality, subjectivity, and (new) constitutional rights, as well as the redefinition of legitimacy, accountability and citizenship in several countries. ^c^ The case studies discussed in the collection vary substantially not only thematically, but also in terms of employed research methods and writing styles. The abundance of discussed issues, arising from the very ambitious objective of the project, at times obscures the rationale behind the book’s structure. Nevertheless, each of the authors highlights the juxtaposition of their analysis with the central themes permeating the whole collection, namely coproduction between science and law, contingency and context-dependence, as well as the relationship between concepts of biopolitics/biopower and the newly coined notions of bioconstitutionalism. Although not all the authors refer to Foucault’s work, it soon becomes obvious that the book is deeply influenced by his theoretical approach. Another visible motif linking all the parts is Sheila Jasanoff’s own body of work to which all authors loyally refer in their contributions. This is to some extent understandable, as Jasanoff (also influenced by Foucault) is one of the pioneers in the field of science and technology studies. However, it also means that the collection is conceptually closed to other sociological and legal theories of Dworkin, Habermas, or Luhmann – to name only a few – that also provide a useful framework for the analysis of the relationship between constitutionalism, medicine, and science. They could significantly enrich the study, especially in its global/transnational dimension. An extensive analysis of these theories extends beyond the scope of this review. Yet, it is perhaps worth mentioning here that for instance, Dworkin’s theory of rights could prove helpful in analysing the relationship between law, politics, and morality, which is at the heart of any constitutional framework (Dworkin 1977, 1997). Habermasian concepts of ‘public sphere’ , ‘deliberation’, and ‘juridification’ have been already present in the public international law and constitutional law discourses for some time now and could also be used to explain the processes taking place in the field of biomedical law (Habermas 1987, 1998, 2003). Finally, an approach rooted in Luhmann’s systems theory provides lenses through which recent developments and debates can be viewed not necessarily as yet another crisis or a systemic failure of regulatory attempts, but on the contrary, as a new phase of regime autonomisation (Luhman, 1993, 1999; Fischer-Lescano 2003, Joerges 2006, Teubner 2012). The notion of spontaneous system self-organisation present in systems theory could have supported some of the co-authors arguments about contingent development of biomedical constitutional law. Undeniably, not every analysis of ‘bioconstitutionalism’ has to engage with these theories. However, acknowledging their existence would benefit the Jasanoff’s collection in that it would necessitate a strong justification for the particular approach that it has adopted and it would have placed it better in the context of constitutional law discourse.

Sheila Jasanoff is not only the editor of the collection but also the author of two substantial chapters. In the introduction she outlines the rationale underlying the volume and its theoretical framework, and explains the central concept of bioconstitutionalism. She starts with the common perception that legal texts have been slow in addressing challenges of the biotechnological revolution instigated by the discovery of the ‘text’ of DNA in 1953. In particular, she points at the reluctance of constitutional lawyers to grapple with new entities such as genes, embryos, stem cells, or hybrids/chimeras. However, she is quick to stress that the main aim of the book is to confront the misconceptions about the modes of cooperation between science and law, and the latter’s delay in responding to scientific developments. All the contributions demonstrate law’s permeability ‘as a conceptual and cultural resource’ preconditioning people’s (and indeed scientists’) ‘normative imageries’. ^d^ Most importantly, she rejects the deterministic view of science in its relationship with law and highlights the influence of legal traditions and cultures – embedded in legal processes, institutions and structures – on biological categorisations. This claim resonates with the well-known concept of ‘interactional coproduction’ of science and politics or law. (Jasanoff 2004, 2005) Thus, one of the central aims of the collection is to demonstrate that two traditionally separated worlds of the normative and the epistemic ‘have supported each other for centuries in patterns of mutual construction, stabilisation, and reinforcement’. ^e^ In this respect the collection seems to confirm the already well-established findings of STS studies.

Therefore, Jasanoff, followed by other authors in the collection, goes beyond this point and argues further that the effects of the recent developments in life sciences on legal institutions such as personhood, rights, citizenship, and legitimacy, have been so profound that they have redrafted established boundaries between science and law, and state and society, and have redefined constitutional frameworks. This is how the notion of bioconstitutionalism emerges as a conceptual continuation of biopower and biopolitics. The term is defined broadly to ‘include full range of sites and processes in which individuals work out their biopolitical relationships with the institutions that regulate them’. It extends beyond the amendments and interpretations of legal texts to include constitutional practices and ‘constitutional moments’ ^f^ which radically restructure state-society relations. Like other constitutional theories, bioconstitutionalism is preoccupied with the questions concerning definition and classifications of rights and loci of decision making power. This broad approach to constitutional law seems to fall into recent discussions in transnational, postnational, and societal constitutional theory, which have also dealt with radical reconceptualisations of constitutionalism. Yet, the book does not elaborate on the contentious question of what constitutes constitutional law. This lack of clear definition of a constitution has to be seen as the biggest weakness of the book, as it remains unclear what makes the events and processes analysed by the authors reach the constitutional threshold and why those rather than other changes contribute to the establishment of ‘bioconstitutionalism’. Instead Jasanoff and other authors ^g^ focus on another crucial motif running through the whole book, namely contingency. Most contributors claim that the science-law relationship and legal structures emerging as a result of scientific changes are to a large degree coincidental in the sense that they are highly dependent on the particular temporal and special context in which they occur.

This is clearly demonstrated in chapters 2 and 3 by A. Wellerstein and S. Jasanoff respectively. Wellerstein’s historic analysis of the sterilisation laws and practices in California in the first half of the 20^th^ century ^h^ leads him to conclude that the adoption and the final abandonment of the programme was not so much about value systems and science-driven ideas about the individual and public health as has been previously suggested. He argues that it was more about decentralization of the administrative and institutional structures which gave enormous decision-making powers to superintendents in mental health institutions and resulted in different numbers of sterilized individuals in particular hospitals across the state. This claim might of course be contested by those who insist that it is ideologies and specific perceptions of mental and public health that enabled the adoption of sterilization laws in the first place, and their strikingly successful implementation. Yet, Wellerstein’s criticism of the normative interpretation and his insistence on the relevance of institutional structures and processes for the effect of the law should not be ignored. Jasanoff’s chapter 3 continues this line of enquiry by showing how constitutional arrangements and frameworks of deliberation have determined legal definitions and moral classifications in the area of embryonic stem cell research. She examines the work of the bioethics committees and councils in the UK, Germany, and the US to show how different perceptions about the role of ethics, rationality, and expertise embedded in institutional designs and legal and political cultures lead to very different legal outcomes in terms of defining new entities brought about by life sciences (embryos, stem cells, human-animal chimeras). These normative classifications have been then internalised, integrated, and perpetuated by scientists. This process of making normative decisions, which Jasanoff calls ‘ontological surgery’, is in her opinion integral to the broader process of ontological politics. In other words, it is not only science that frames the legal discourse, but it is also politics, law and bioethics that shape science. The comparative perspective employed by Jasanoff is aimed to reemphasise inherent differences in the professional bioethical discourse, which in her opinion, has been universalised too quickly.

Chapters 4 and 5 written by G. Testa and I. Metzler further explore these arguments. In his contribution Testa also employs the comparative method of analysis to investigate how cloned cells were enabled as socially legitimate scientific objects in Italy, the UK and the US. Through the analysis of the House of Lords’ decision in the *Quintavalle* case ^i^, the proceedings of the Italian Dulbecco Commission and the proposal of the US President’s Council on Bioethics he demonstrates how different modes of coproduction between ‘the epistemological, the ontological and the normative’ ^j^ lead to very different outcomes in terms of legalisation of the somatic cell nuclear transfer and ‘how political cultures (their historical constraints, their discursive resources, and their ways of distributing and recognising expertise) are integral to the development of technoscientific objects’. ^k^ However, as shown by I. Metzler in her chapter on the Italian embryonic stem cell debate, it can also be the inactivity of citizens – who did not use their constitutional right to vote in a referendum – rather than any particular activity of the state that transforms embryos into legal persons. ^l^ This conclusion might already seem well known to those who have interest in the area of reproductive medicine. However, Metzler provides a detailed analysis of the events that preceded and followed the Italian referendum on IVF and stem cell research and presents a meticulous depiction of the complex triangular interactions between the Catholic Church, Italian voters, and the state.

The next part of the book, although not formally divided, moves away from the discussions about new biological entities. In chapters 6 and 7 J.D. Aronson and D.E. Winickoff insightfully analyse the ways in which the use of forensic DNA testing ^m^ and biobanks in the U.S. enable the creation and redefinition of constitutional rights, such as the right to postconviction testing, and the freedom from unreasonable searches and seizures guaranteed by the Fourth Amendment. Aronson demonstrates that the possibility of performing a DNA test long after the conclusion of a trial leads to a conflict between two main legal principles, the doctrine of finality of courts’ judgments (i.e. social order) and the doctrine of certainty of evidence and court procedures (i.e. the fundamental principle of a fair trial). His historical analysis of the US Supreme Court jurisprudence reveals that the mechanisms and rationale behind the failure to construct a constitutional right to postconviction DNA testing depended as much on policy considerations as on the reluctance of the justice system to accept DNA testing as a ‘revelation machine’ despite the unprecedented raise of its status. The power of ‘imaginaries of technology’ ^n^ in the judicial decision making process constitutes the centre of Winickoff’s chapter. It shows how different perceptions of technologies influenced the decisions about constitutionality of large-scale forensic DNA databases and the construction of special categories of subjects (convicted felons) who in the courts’ view have lower expectation of privacy, and ‘whose rights are reframed without their direct participation’. ^o^

The last part of the book is concerned with different coproduction processes involved in legalisation of new technologies, the (re)conceptualisation of persons or groups subjected to these technologies, and their rights in different parts of the world. In chapter 8 M. Tallachchini interestingly describes the distinct regulatory approaches to risks associated with xenotransplantation. She shows how they stem from distinct visions of society: contractual and liberal vision in the US and paternalistic and vertical vision in Europe. ^p^ Similarly, K.S. Rajan in his chapter 9 convincingly argues that the construction of subjectivity in contemporary global biomedicine is deeply engrained in regimes of value and specific histories of ‘biocapital’. Using two examples of biotech companies in the US and India, he shows how genetic technologies and industries in the US view patients as consumers of genetic testing subjected to perpetual possible consumption, while in India the state acts as a market agent setting up their premises of biotech firms in areas of high unemployment, thus making the Indian population available as experimental subjects to Western corporate interests and in turn perpetuating postcolonial inequities. ^q^

Chapters 10, 11, 12 by the end of the book investigate the effects that new technologies have on the notion of democracy, legitimacy and citizenship. For J. Reardon (Ch. 10) the struggle to avoid labelling of socially and scientifically constructed groups of research subjects involved in the Human Genome Diversity Project and the HapMap project constitutes a constitutional moment which requires a response that would take into account the dynamics of coproduction. ^r^ For R. Doubleday and B. Wynne (Ch. 11) the unprecedented public engagement in the UK GMO debates on commercialisation and promotion, and the public’s attempts to influence the decision making processes, also illustrate developments in the constitutional understanding of science, agency, and citizens’ rights in relation to the state. ^s^ Finally, taking these considerations on to the supranational level J. Dratwa (Ch. 12) looks at the text and *travaux preparatoire* of the European Parliament resolution on the precautionary principle, and concludes that its elevation to a constitutional principle was used to support the unity of purpose among the EU institutions, but also to strengthen the legitimacy of the EU in relation to member states. ^t^ While especially the first part of the argument is well known to those engaged in the regulation of plant and human genetics, the second part is more interesting and should have been further developed to include more recent and/or potential future advancements in the area.

The research and findings presented in this collection are extremely informative, especially for constitutional and comparative lawyers interested in the processes of constitutionalisation who are less familiar with the literature on coproduction. At the same time, however, they might find parts of the analysis difficult to engage with. This is primarily because of the rather vague concept of constitution assumed rather than developed throughout the book. Although Jasanoff underlines the breadth of the notion of bioconstitutionalism in her introduction, she does not refer to any constitutional theory that could provide a comparison for her definition. It would seem that that the main implicit point of reference for Jasanoff is the U.S. constitution coupled with the Supreme Court’s jurisprudence, which would suggest a rather narrow and traditional understanding of constitution. However, this remark must remain a speculation. Apart from K.S. Rajan, none of the authors attempts to explain their understanding of constitutional framework against which they assess their case studies. This, of course, allows for flexibility in the scope and depth of analysis, and is to some extent understandable at the beginning of such an ambitious and large project that certainly exceeds one book. However, it also undermines the full potential of this particular collection. Globalisation has transformed the meaning of notions such as sovereignty or constituting and constitutive power and as a result theories of constitutionalism have undergone dramatic changes. ^u^ The epistemic and normative developments described in the collection affect and are affected by these movements and it is unfortunate the book has not acknowledged these dynamics more explicitly. Had it done so, it might have alleviated the impression that the collection is still more about coproduction of knowledge and law and less about bioconstitutionalism. This impression might stem from the fact that the authors seem to employ a very broad (or maybe divergent?) understanding of law that includes not only constitutional and statutory rights, judicial decisions, but also soft law instruments and outcomes of deliberative processes. Such an understanding is acceptable and common in sociological, science and technology, and even some legal studies. However, it does carry the risk of blurring semantic and normative boundaries. Clarifying the distinctions between law and non-law, as well as law and constitutional law, would help address a potential argument suggesting that, as indeed discussed in one of the chapters ^v^, courts and legislators are slow in acknowledging new constitutional rights. It would reinforce the significant and apposite claim about the constitutional changes initiated by life sciences and the constitutionalisation of biomedical law. None of this should be taken as being dismissive or overly critical towards the book. Rather it is a call for desperately needed further investigation in this area. The collection edited by S. Jasanoff is a very promising beginning of a long journey on which lawyers should finally embark.

## Endnotes

^a^ It has been initiated at a panel discussion at the 2010 annual meeting of the Society for Social Studies of Science in Tokyo.

^b^ Although S. Jasanoff graduated as a J.D. from Harvard Law School she is most famous for her work as a professor in Science and Technology Studies. Also D.E. Winickoff is a Harvard Law School graduate who moved to the area of STS studies.

^c^ The research includes case studies from Australia, Canada, Germany, India, Italy, the EU, the UK, and the U.S. with a heavier focus on the last two.

^d^ Introduction, 9.

^e^ Introduction, 13.

^f^ S. Jasanoff refers to the term developed by Bruce Ackerman in 1983. Introduction, 10.

^g^ With the exception of M. Tallacchini, J. Dratwa, and K.S. Rajan, who in chapters 8, 12, and 9 accordingly deal with the supranational and global aspects of bioconstitutionalism. However, they do not expressly address the problem of what constitutes law (in particular constitutional law), and seem to operate within certain assumptions and broad definitions of law.

^h^ ‘Between 1909 and 1950s the state of California authorised over 20.000 sterilisations of mentally ill and mentally deficient patients.’, Chapter 2: A. Wellerstein, ‘States of Eugenics: Institutions and Practices of Compulsory Sterilization in California’, note 1 above, 29–58, 29.

^i^ R (on the application of Quintavalle) v Secretary of State for Health [2003] UKHL 13.

^j^ G.Testa, More than Just a Nucleus: Cloning and the Alignment of Scientific and Political Rationalities, 85–104, 102.

^k^ Ibid.

^l^ I. Metzler, Between Church and State: Stem Cells, Embryos, and Citizens in Italian Politics, 105–124.

^m^ J.D. Aronson, Certainty v. Finality: Constitutional Rights to Postconviction DNA Testing, 125–146.

^n^ D.E. Winickoff, Judicial *Imaginaries of Technology*: Constitutional Law and the DNA Round Up, 147–168.

^o^ Ibid., 165.

^p^ M. Tallacchini, Risks and Rights in Xenotransplantation, 169–192.

^q^ K. S. Rajan, Two Tales of Genomics: Capital, Epistemology, and Global Constitutions of the Biomedical Subject, 193–216.

^r^ J. Reardon, Human Population Genomics and the Dilemma of Difference, 217–238.

^s^ R. Doubleday and B. Wynne, Despotism and Democracy in the UK: Experiments in Reframing Citizenship, 239–262.

^t^ J. Dratwa, Representing Europe with the Precautionary Principle, 263–286.

^u^ There is an impressive and insightful body of literature in this area: Ch. Joerges and E. Petersmann. 2006. Constitutionalism, Multilevel Trade Governance and Social Regulation, Oxford: Hart Publishing; M.Loughlin, N.Walker. 2007. The Paradox of Constitutionalism: Constituent Power and Constitutional Form. Oxford: OUP; N. Walker. 2008. Beyond Boundary Disputes and Basic Grids: Mapping Global Disorder of Normative Orders. International Journal of Constitutional Law, 6: 373–396; J. Klabbers, A. Peters, G. Ulfstein (eds.). 2009. The Constitutionalization of International Law. Oxford: OUP; N. Krisch. 2010. Beyond Constitutionalism. The Pluralist Structure of Postnational Law, Oxford: OUP; P. Dobner, M. Loughlin (ed.) 2011.Twilight of Constitutionalism, Oxford: OUP; C. Thornhill. 2011. A Sociology of Constitutions: Constitutional and State Legitimacy in Historical-Sociological Perspective. Cambridge: CUP; G. Teubner 2012. Constitutional Fragments. Societal Constitutionalism and Globalisation, Oxford: Oxford University Press; G.W Anderson. 2012. Beyond `Constitutionalism Beyond the State. Journal of Law and Society 39:3: 359–83.

^v^ J.D. Aronson, Certainty v. Finality: Constitutional Rights to Postconviction DNA Testing, 125–146.
